# Pituitary Apoplexy After a Major Abdominal Surgery: A Case Report

**DOI:** 10.7759/cureus.52966

**Published:** 2024-01-25

**Authors:** Abdulaziz M Alshahrani, Ali Al Bshabshe, Mohammed B Al Shahrani

**Affiliations:** 1 Otolaryngology-Head and Neck Surgery, Aseer Central Hospital, Abha, SAU; 2 Intensive Care Unit, Aseer Central Hospital, Abha, SAU; 3 Medicine, King Khalid University, Abha, SAU

**Keywords:** major abdominal surgery, hepatic hydatid cyst resection, severe headache, pituitary adenoma, pituitary apoplexy

## Abstract

Pituitary apoplexy is a major complication of pituitary adenoma, and the diagnosis might be challenging if the patient presents with signs of meningeal irritation or electrolyte imbalance. It can be fatal if not diagnosed and treated appropriately. Apoplexy is the first clinical presentation in the majority of pituitary adenoma cases. The pathophysiology of pituitary apoplexy involves bleeding and/or ischemia of pituitary enlargement. In this case report, we present a case of pituitary apoplexy that developed after a major abdominal surgery. The patient presented with headache, hypertension, and visual loss. After confirming the diagnosis through a CT scan, the patient underwent a transsphenoidal surgical decompression.

## Introduction

Pituitary apoplexy is a rare complication of pituitary adenoma characterized by the sudden onset of severe headache, vision affection, cranial nerve palsy, and/or hypopituitarism caused by bleeding or ischemia of the pituitary tumor [1,2]. It has a serious impact and a probable fatality [3]. The well-known risk factors include pregnancy, high intracranial pressure, hypertension, major surgery, and the use of anticoagulants [3]. The management of pituitary apoplexy generally depends on the presenting symptoms, although most authors are in favor of early surgical decompression [4].

## Case presentation

Here, we present a 52-year-old man who had no known chronic diseases before. He was admitted to the inpatient department with a large hydatid cyst and underwent an open right hepatectomy. During the surgery, he lost a considerable amount of blood and was transfused with one unit of packed red blood cells. Upon admission to the intensive care unit, the patient was already extubated in the operation room. He was conscious, oriented, and alert. His Glasgow Coma Scale (GCS) score was 15/15, with vital signs within normal ranges.

On postoperative day 1, the patient started to experience a severe headache, high blood pressure, and left-eye vision loss. The patient had no history of decreased level of consciousness, seizure, numbness, weakness, trouble speaking, or difficulty understanding speech. The systemic review was unremarkable. On examination, he was conscious, oriented, and alert. His GCS was 15/15, with vital signs as follows: blood pressure 142/77, heart rate 85, temperature 37.5, respiratory rate 24, and oxygen saturation 97% on room air. Neurological examination was unremarkable apart from left-eye vision loss and right-eye temporal hemianopia. His complete blood count, coagulation profile, and electrolytes were within normal ranges.

**Figure 1:**
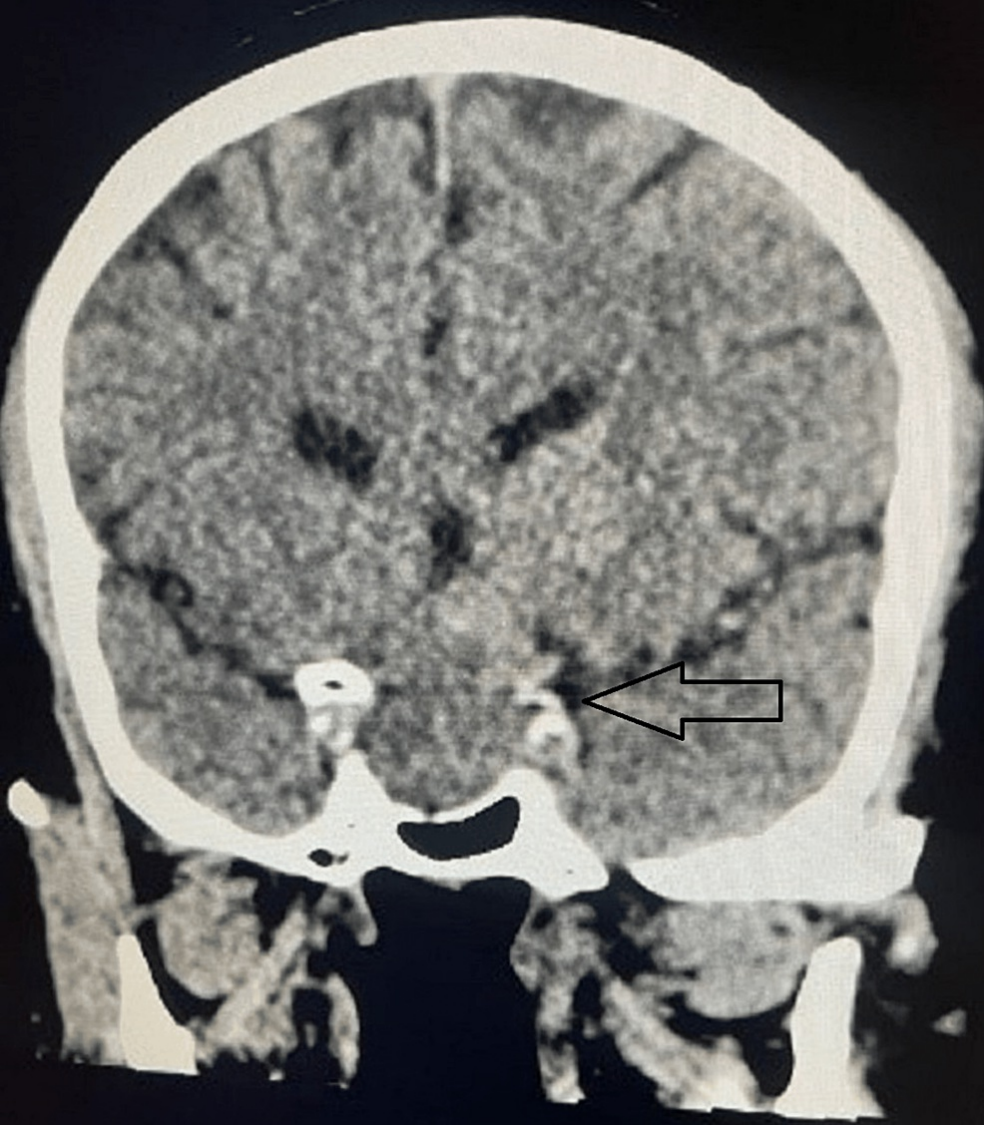
CT scan coronal view showing sellar and suprasellar mass lesions.

**Figure 2:**
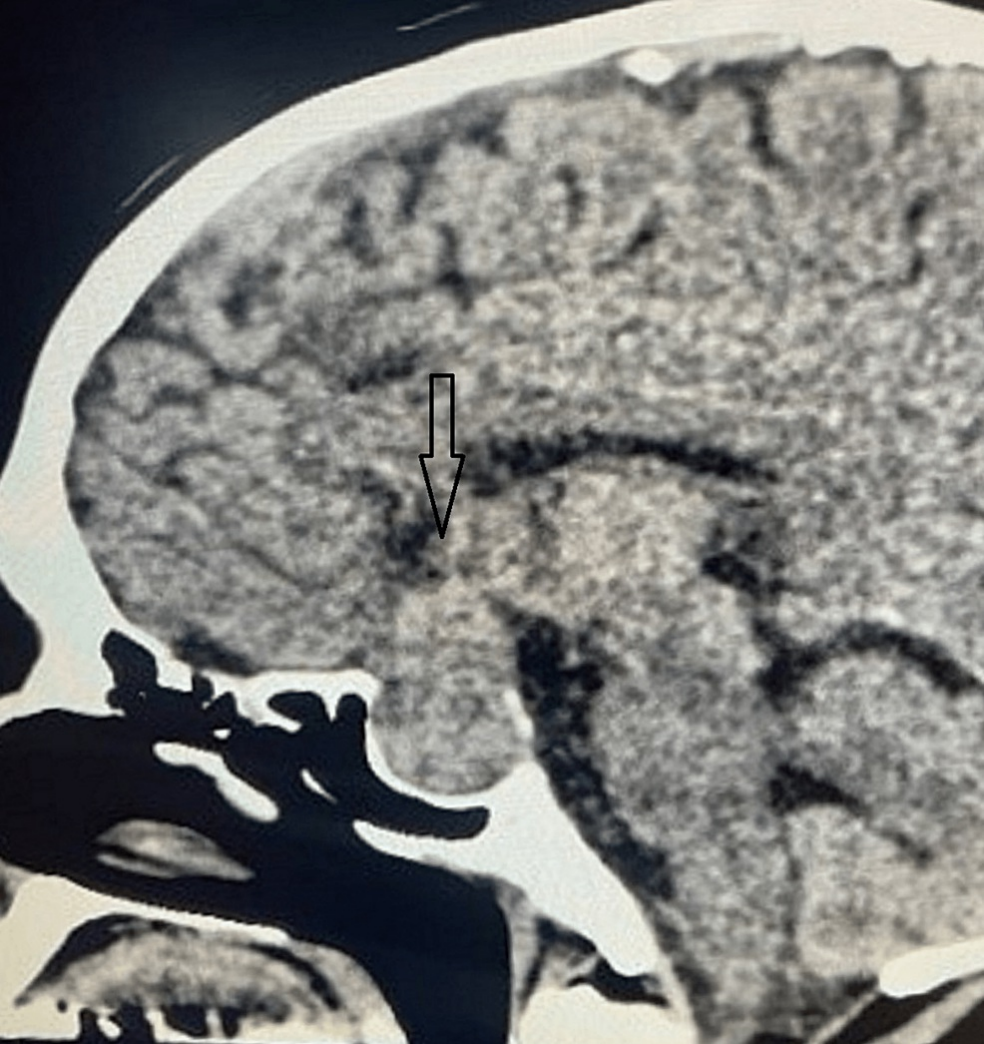
CT scan sagittal view showing sellar and suprasellar mass lesions.

The CT scan showed well-defined, relatively hyperattenuating, homogeneous sellar and suprasellar mass lesions (Figures 1, 2), mildly expanding the sella and effacing the suprasellar cistern. The remodeling of the sella is noted with mild calcification. There was no evidence of intracranial hemorrhage, acute transcortical territorial infarct, cerebral arterial occlusion, or venous thrombosis. With the confirmed diagnosis of pituitary apoplexy, the patient was started on steroids and underwent transsphenoidal surgical decompression. The surgery went uneventfully, with immediate improvement in headache and partial recovery of visual disturbances.

## Discussion

Pituitary apoplexy is a major complication of pituitary adenoma, and its diagnosis might be challenging if the patient presents with signs of meningeal irritation or electrolyte imbalance [5]. It can be fatal if not diagnosed and treated appropriately. Apoplexy is the first clinical presentation in the majority of pituitary adenoma cases, as in this case the patient was not diagnosed before [6]. The pathophysiology of pituitary apoplexy involves bleeding and/or ischemia of pituitary enlargement [7]. The precipitating factors of this condition have been discussed in the literature, including pregnancy, high blood pressure, major surgery, use of anticoagulants or antiplatelets, and high intracranial pressure. In this case, multiple risk factors were identified, including major surgery, blood loss in the operating room, and high blood pressure.

In our review of the literature, we found that apoplexy is usually reported after cardiac surgery. However, a few cases have been reported after abdominal surgeries, and to the best of our knowledge, none of them have been reported after hydatid cyst resection [8]. Moreover, apoplexy was reported in most of the cases after major surgery; however, it was reported after routine surgery without any significant hemodynamic disturbances [9]. Pregnancy is a well-known risk factor for the development of pituitary apoplexy and is related to hormonal stimulation and high blood flow during pregnancy [10]. Additionally, cases of pituitary apoplexy have been reported post-COVID-19 vaccination as a consequence of vaccine-induced thrombophilia-thrombocytopenia (VITT) syndrome or autoimmune/autoinflammatory syndrome induced by adjuvants (ASIA) syndrome [11,12].

In this case, the patient experienced left-eye vision loss and right temporal hemianopia, which can be explained by asymmetrical pressure on the optic chiasm and optic nerve. Ophthalmoplegia and papilledema are signs that direct the diagnosis toward acute, expanding mass; however, they can be absent, as in the current case [8]. After prompt diagnosis, the management starts with stabilizing the patient hemodynamically, correcting electrolytes, and administering steroids. The surgical stress and the significant blood loss during the surgery are likely to be the precipitating factors for the development of pituitary apoplexy in this case.

The management of pituitary apoplexy depends on the patient's symptoms, whether conservative or surgical. Both modalities have been used and advised in the literature, but there is no strong evidence-based recommended modality. However, surgical management is usually chosen when there is an impaired level of consciousness, decreased visual acuity, or visual field defect [13]. It has been reported that early surgical intervention by transnasal transsphenoidal resection is associated with better outcomes regarding vision [14]. Conservative management requires close monitoring of the patient for any deterioration or hormonal imbalances, which might indicate long-term hormonal replacement. This case report highlights the importance of suspecting pituitary apoplexy after any surgery, especially if the surgery is associated with a considerable amount of blood loss or hemodynamic instabilities. It emphasizes the importance of utilizing imaging, such as a CT scan, when the patient presents with the aforementioned symptoms.

## Conclusions

In conclusion, this is an old adult who underwent major abdominal surgery. On the following day, he experienced headache, hypertension, and vision problem. The diagnosis was confirmed by a brain CT scan as pituitary apoplexy and was managed surgically. The surgery was not eventful, and the patient was discharged home to be followed in the outpatient department. Pituitary apoplexy is a rare complication of pituitary tumor that can happen after major surgery. Physicians should be vigilant to suspect this condition as one of the postoperative complications of major surgeries. A multidisciplinary team for the management of such cases, particularly when there is no strong evidence-based approach, is highly beneficial.
